# Supplementary material for: Central American and Caribbean Consensus Document for the Optimal Management of Oral Anticoagulation in Patients with Non-Valvular Atrial Fibrillation Endorsed by the Central American and Caribbean Society of Arterial Hypertension and Cardiovascular Prevention
Source: J Clin Med. 2024 Jan 5;13(2):314. doi: 10.3390/jcm13020314 (PMC10816126; doi:10.3390/jcm13020314)
Supplement: Supplementary file 1 [file jcm-13-00314-s001.zip › jcm-2735260-supplementary.pdf]

Figure S1. Considerations that, according to the panelists, should be taken into account for the implementation and the follow-up of the Delphi statements as recommendations in their country.

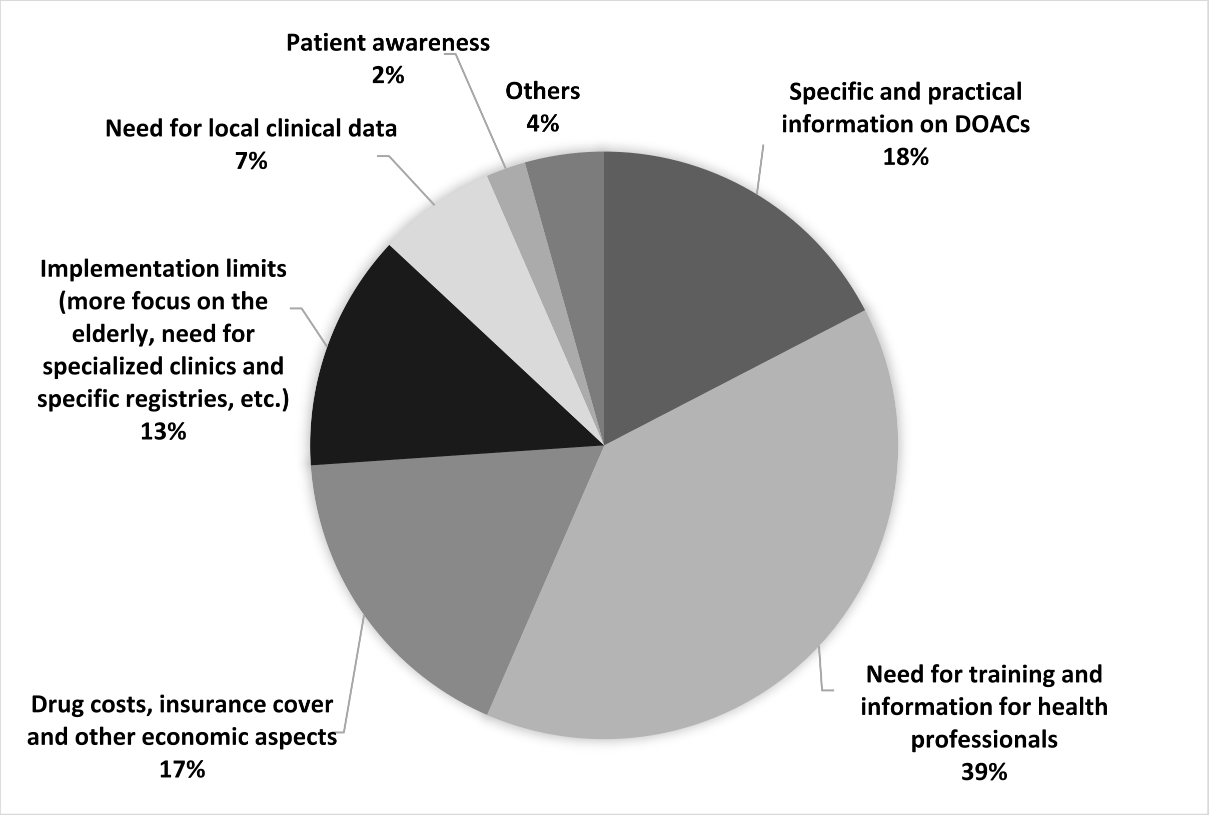

Figure S2. Actions to implement to facilitate the use and access to DOACs in the Central America and Caribbean area.

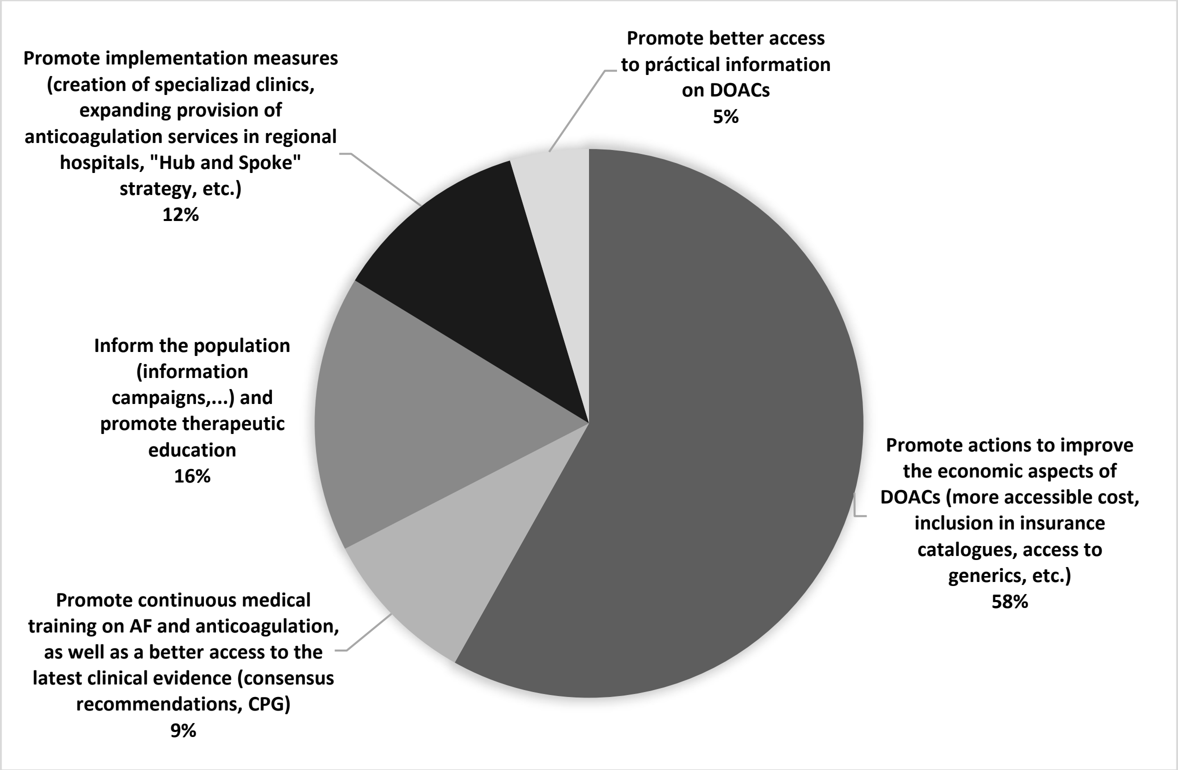

Table S1. Sources of information

**Databases**	• Large bibliographic databases:<br>o PubMed/MEDLINE with the use of corresponding methodological filters<br>o Tripdatabase<br>• Clinical practice guideline repositories:<br>o G-I-N International Guideline Library<br>o GuíaSalud<br>• Repositories of guidelines from prominent European and American cardiology societies such as NICE, SIGN, AHA.

Table S2. Search strategy, specific filters, key keywords

**Temporal limits**	• Last 5 years.
**Population**	• NVAF patients.
**Intervention**	• Eligible interventions will include oral anticoagulant treatment, including vitamin K antagonists (VKAs) such as warfarin and direct oral anticoagulants (DOACs).
**Lenguage**	• Spanish<br>• English
**Publication type**	• Priority 1: Clinical practice guidelines.<br>• Priority 2: Consensus documents.<br>*"The systematic literature search will begin with Priority 1, focusing on clinical practice guidelines. In a second step, if there are not enough results or if the obtained results do not address the specific question, the search will be expanded to Priority 2, including consensus documents."*
**Title filters**	*We will prioritize the retrieval of references based on the fields of title or abstract and descriptors in case the volume of literature retrieved is notably excessive when using free text.*
**Key words (language Pubmed/MEDLINE)**	• Patient: ("Atrial Fibrillation"[Mesh] OR "non-valvular atrial fibrillation"[Title/Abstract] OR "atrial fibrillation nonvalvular"[Title/Abstract] OR "non valvular atrial fibrillation"[Title/Abstract])<br>• Oral Anticoagulation: ("Anticoagulants"[Mesh] OR "Anticoagulants" [Pharmacological Action] OR "oral

	anticoagulation [Text Word] OR anticoagulation[Title/Abstract])<br>o VKAs: "antivitamins K" [Supplementary Concept] OR "Warfarin"[Mesh] OR "vkas"[Title/Abstract] OR "vitamin k antagonists"[Title/Abstract] OR "Acenocoumarol"[Mesh]<br>o DOACs: "non vka oral anticoagulant"[Title/Abstract] OR “nonvitamin-K-antagonist oral anticoagulant”[Text Word] OR "nonvitamin k antagonist oral anticoagulants"[Text Word] OR "noac"[Text Word] OR "Dabigatran"[Mesh] OR "Rivaroxaban"[Mesh] OR "apixaban" [Supplementary Concept] OR "edoxaban" [Supplementary Concept] OR "direct oral anticoagulants"[Title/Abstract] OR "doac"[Title/Abstract]<br>o Bridging therapy: heparin bridge therapy[Text Word]
